# Botulinum Toxin and Deep Brain Stimulation in Dystonia

**DOI:** 10.3390/toxins16060282

**Published:** 2024-06-20

**Authors:** Julia Carvalhinho Carlos de Souza, Ananda Carolina Moraes Falcone, Renata Montes Garcia Barbosa, Miriam Carvalho Soares, Renato Munhoz, Marina Farah, Tamine Capato, Sara Carvalho Barbosa Casagrande, Marcela Ferreira Cordellini, Gabriel de Castro Micheli, João Carlos Papaterra Limongi, Egberto Reis Barbosa, Clarice Listik, Rubens Gisbert Cury

**Affiliations:** 1 Movement Disorders Center, Department of Neurology, School of Medicine, University of São Paulo, São Paulo 05403-000, Brazil; ananda.falcone@alumni.usp.br (A.C.M.F.); renatamontesgarcia@hotmail.com (R.M.G.B.); miriam.carvalho@ufpe.br (M.C.S.); taminec@usp.br (T.C.); sara.casagrande@hc.fm.usp.br (S.C.B.C.); joao.papaterra@hc.fm.usp.br (J.C.P.L.); egbertob@8415.com.br (E.R.B.); clarice.listik@hc.fm.usp.br (C.L.); rubens_cury@usp.br (R.G.C.); 2 Morton and Gloria Shulman Movement Disorders Centre and the Edmond J. Safra Program in Parkinson’s Disease, Toronto Western Hospital, University Health Network, Toronto, ON M5T 2S8, Canada; renata.garcia@hc.fm.usp.br; 3 Cajuru University Hospital, Pontíficia Universidade Católica do Paraná, Curitiba 80050-350, Brazil; ma.mafarah@hotmail.com; 4 Department of Neurology, Radboud University Medical Center, 6525 Nijmegen, The Netherlands; 5 Instituto de Neurologia de Curitiba, Curitiba 81210-310, Brazil; macordellini@gmail.com; 6 Department of Neurology, University Hospital Clementino Fraga Filho, Federal University of Rio de Janeiro, Rio de Janeiro 21941-617, Brazil; gcmicheli@yahoo.com.br; 7 Hospital Israelita Albert Einstein, São Paulo 05652-900, Brazil

**Keywords:** botulinum toxin, dystonia, Deep Brain Stimulation, abobotulinum toxinA, onabotulinum toxinA

## Abstract

Deep Brain Stimulation (DBS) is a recognized treatment for different dystonia subtypes and has been approved by the Food and Drug Administration (FDA) since 2003. The European Federation of Neurological Societies (EFNS) and the International Parkinson and Movement Disorders Society (MDS) recommend DBS for dystonia after failure of botulinum toxin (BoNT) and other oral medications for dystonia treatment. In addition, several long-term studies have demonstrated the continuous efficacy of DBS on motor and quality of life (QoL) scores. However, there are only a few reports comparing the overall impact of surgical treatment in BoNT protocols (e.g., dosage and number of selected muscles before and after surgery). This retrospective multicenter chart-review study analyzed botulinum toxin total dosage and dosage per muscle in 23 dystonic patients before and after DBS surgery. The study’s primary outcome was to analyze whether there was a reduction in BoNT dosage after DBS surgery. The mean BoNT dosages difference between baseline and post-surgery was 293.4 units for 6 months, 292.6 units for 12 months, and 295.2 units at the last visit. The median total dose of BoNT in the preoperative period was 800 units (N = 23). At the last visit, the median was 700 units (*p* = 0.05). This represents a 12.5% reduction in BoNT median dosage. In conclusion, despite the limitations of this retrospective study, there was a significant reduction in BoNT doses after DBS surgery in patients with generalized dystonia.

## 1. Introduction

Dystonia is a movement disorder characterized by muscle contractions that can be sustained or intermittent and can cause abnormal movements and postures. It has varied pathophysiology and various phenomenologies. Regarding etiology, it can occur secondary to many acquired and inherited etiologies, or it can be idiopathic. Dystonia can also be presented with a variable body distribution and can be divided into two different axes: clinical features and etiology [1,2].

Clinical treatment varies according to severity, age, type, and body distribution. Treatment may include oral medications such as anticholinergics, baclofen, and benzodiazepines. Additionally, intramuscular botulinum toxin (BoNT) type A injections are the first-line treatment for primary cranial and cervical dystonia and writing dystonia. It can also be used in segmental and generalized dystonia, although attention to dose limit is needed [1,3,4]. However, these interventions often fail to provide adequate symptom relief or may be limited by various side effects [1].

Several studies have demonstrated a sustained improvement in the motor and in the quality of life (QoL) scores for dystonia and disability after deep brain stimulation (DBS) surgery. The major indications for DBS for dystonia include genetic and drug-induced dystonia refractory to pharmacological treatment [5,6,7]. There are some factors that can contribute to variability in post-surgical outcomes, including duration of the disease, phenomenology of the movement, dystonia classification, body distribution, orthopedic deformity, etiology, and genetic subtypes [7]. In this way, the European Federation of Neurological Societies (EFNS) and the International Parkinson and Movement Disorders Society (MDS) suggest considering DBS after failure of first-line treatments in dystonic patients with significant disability [8,9,10].

In the literature, there are few studies that have evaluated the usage of botulinum toxin before and after DBS surgery in dystonic patients [11,12,13]. One retrospective study from 2022 analyzed nine patients with cervical dystonia and found a reduction in BoNT dose after DBS in four patients, with an average of 22% dose reduction [11]. Another study demonstrated that in a case series of three patients with generalized dystonia who underwent DBS surgery, preoperative BoNT dosages and those after the procedure were not different [12].

In order to better analyze the association between DBS and BoNT and to verify DBS’s impact on the BoNT dosage injections in patients with generalized dystonia, a multicentric analysis of twenty-three pre-and postoperative BoNT protocols was performed.

## 2. Results

The patients’ demographic data are shown in Table 1. The patients’ ages ranged from 16 to 55 years, with a mean age of 37 years. The time since surgery varied from between two and seven years, with an average of 4.3 years. Disease duration ranged from 9 to 38 years, with an average of 24 years. Eight of the patients had symptoms in childhood and adolescence. The patients had an age range of 4–44 years at symptom onset. The baseline patient characteristics are summarized in Table 1.

Of the 23 patients evaluated, seven had confirmed genetic disorders. The most common genes were *DYT-THAP1* and *DYT-TOR1A*, which were present in two patients each. Single cases of *DYT-PRKRA*, *DYT-SGCE*, Mohr–Tranebjaerg, PKAN, tardive dystonia, and perinatal hypoxia were observed. The other 13 patients were not tested or had negative results (four patients were tested for *DYT-THAP1* and *DYT-TOR1A*, with negative results).

Patients were referred to DBS surgery due to refractory generalized dystonia despite the best medical care with oral medications and botulinum toxin. The prescriptions included benzodiazepines, cyclobenzaprine, baclofen, trihexyphenidyl, and biperiden. Oral medications and botulinum toxin application were adjusted according to the patient’s clinical evolution.

The DBS targets were chosen based on individual clinical characteristics and each center’s preference. Six patients underwent bilateral subthalamic nucleus (STN) DBS surgery. Five of those patients were part of a research protocol and were randomized to undergo STN DBS. The other patient first underwent pallidotomy. However, it was decided to perform rescue STN DBS after clinical failure and progression of dystonia. The remaining seventeen patients underwent bilateral globus pallidus internus (GPi) DBS.

The most commonly used botulinum toxin was abobotulinum toxin. Two patients received onabotulinum in the first application, with no subsequent use of onabotulinum. Eight other patients used onabotulinum for all applications. The major explanation for this difference was hospital toxin availability. Muscle selection was based on the most bothersome symptom, which included the cervical, arm, leg, and axial muscles. All but one patient had cervical muscles included before surgery. Two of these patients (8.69%) developed remission of cervical dystonia, and the rest maintained the same protocol of muscle selection. Six patients (26.08%) had axial muscles selected prior to surgery, with a reduction to two patients in the long term (8.69%). One of these patients had a documented dose reduction of 50%.

As shown in Table 2, the median total dose of BoNT in the preoperative period was 800u (N = 23). After surgery, the medians were as follows: 6 months (N = 17): 600u; 12 months (N = 21): 640u; current dose (N = 20): 700u (*p* = 0.05). At the end of the study period, there was a 12.5% reduction in median dosage. Data regarding clinical correlations of the BoNT dose reduction and dystonia rating scales were not available in patients’ medical charts.

The mean differences between baseline and post-surgery dosages (6 months, 12 months, and current dose) were 293.4, 292.6, and 295.2, respectively. There were no significant differences between each of the post-surgery doses (*p* > 0.999; Table 3).

**Figure 1:**
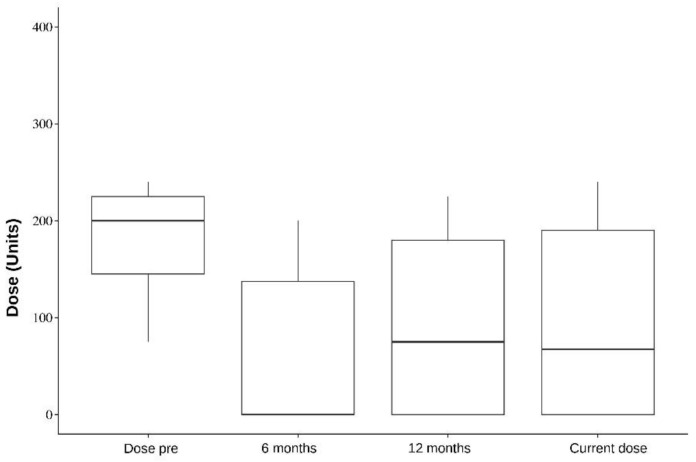
Splenius capitis. Dose per period.

**Figure 2:**
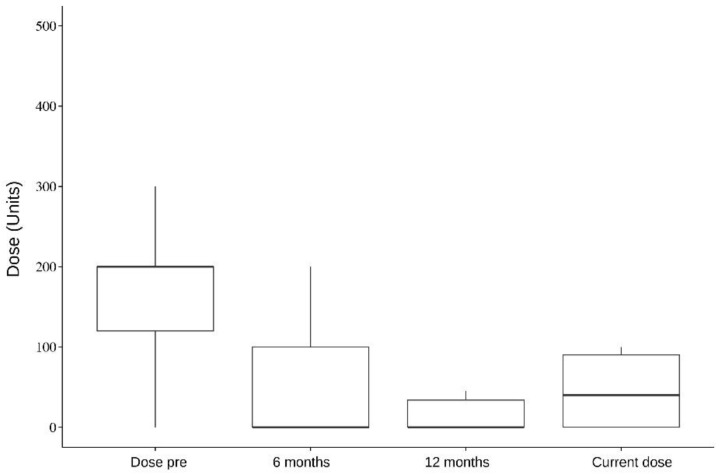
Paravertebral muscles. Dose per period.

**Figure 3:**
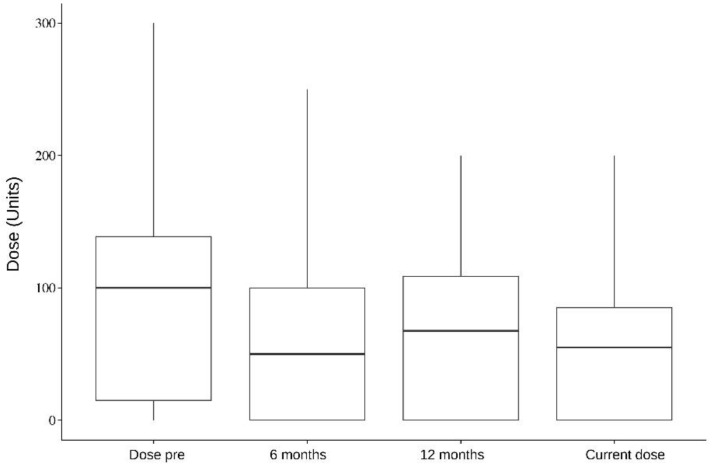
Sternocleidomastoid. Dose per period.

**Figure 4:**
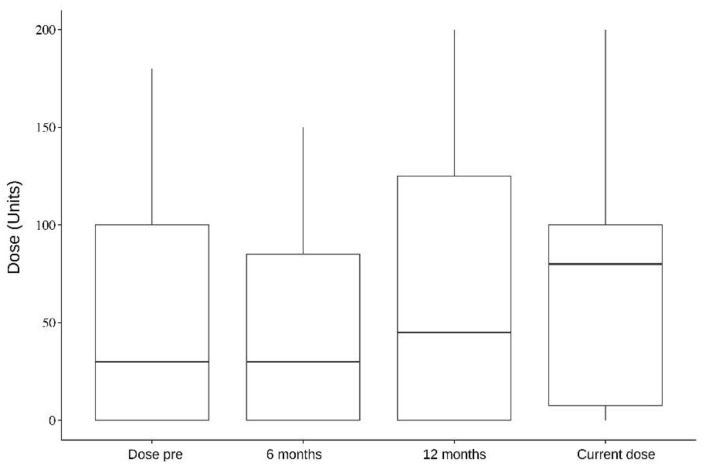
Semispinalis. Dose per period.

**Figure 5:**
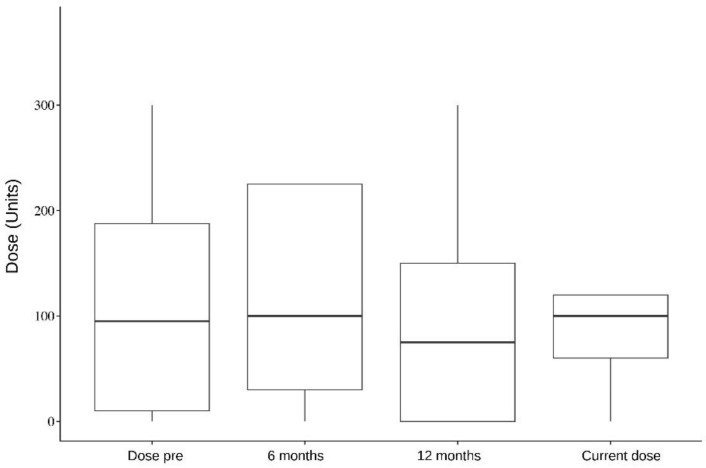
Trapezius. Dose per period.

In addition, five muscles were analyzed individually: the splenius capitis, paravertebral, sternocleidomastoid, semispinalis, and trapezius. Splenius capitis analysis showed a mean difference between pre-surgery and 6 months dose of 126.5; pre-surgery and 12 months dose of 118; and pre-surgery and current dose of 119.1, which were statistically significant (*p* < 0.001). Paravertebral muscles analysis, on the other hand, showed a mean difference between pre-surgery and 6 months dose of 106.6 (*p* = 0.087, not statistically significant); pre-surgery and 12 months dose of 124 (*p* = 0.009); and pre-surgery and current dose of 119.1 (*p* = 0.003). The data of all the analyzed muscles are presented in Table 4, Table 5, Table 6, Table 7 and Table 8 and Figure 1, Figure 2, Figure 3, Figure 4 and Figure 5.

## 3. Discussion

Although there are many reasons why botulinum toxin dosage in generalized dystonic patients could require an increase in dosage with time (disease progression, DBS battery discharge, technical limitations, and, rarely, immunogenicity for BoNT), the study found a reduction in BoNT doses after DBS.

The mean dose reduction comparing patients before DBS surgery with 6 months after surgery, 12 months after surgery, and the last BoNT session were 293.4, 292.6, and 295.2, respectively. No significant reduction was verified comparing between dosages at 6 months, 12 months, and the last BoNT session.

The main conclusion of this analysis is that DBS treatment for generalized dystonia seems to result in stable improvement in dystonic contraction. Although no physical examination data and dystonia rating scales were included in this study, this is a possible conclusion, considering that the objective of the treatment is to reduce muscle contractions to recover functionality. Improvement after DBS surgery not only reduced muscle contractions to a level where they were better controlled with current treatment but also reduced the dose of the previous botulinum toxin treatment.

In a separate muscle analysis, only splenius capitis showed a statistically significant dose reduction in all time periods after DBS compared with the pre-surgery dose. The mean reduction compared with the pre-DBS dose was 126.5 at 6 months, 118 at 12 months, and 119.1 with the last dose (<0.001). Paravertebral showed a statistically significant dose reduction in two time periods: pre-surgery and 12 months dose, which showed a mean reduction of 124 (*p* = 0.009); and pre-surgery and current dose, with a mean reduction of 119.1 (*p* = 0.003). The trapezius, sternocleidomastoid, and semispinalis muscles showed a clear reduction in doses before and after DBS. Still, they were not statistically significant, which might be due to our small sample size. This improvement could point towards a specific set of muscles where improvement of DBS therapy may be better.

This observation also applies to the various body segments. There was a marked reduction in the axial and cervical areas, with a simultaneous increase in the appendicular and orofacial dosage. This observation might lead to the conclusion that DBS therapy is responsible for greater improvement in axial symptoms and not so much in the orofacial and appendicular regions. Therefore, to achieve maximum symptom relief, a greater dose of toxin may be used in these areas. However, this observation must be interpreted with caution due to the limitations of our study.

Few studies have investigated the effects of DBS on body segments. One study included 18 patients with dystonia related to KMT2B mutations and reported greater improvements in motor function in patients with trunk and cervical dystonia, with less clinical impact in patients with laryngeal dystonia [13]. This conclusion is similar to that of this study. Therefore, this study supports the results that DBS improves certain segments more than others, and these data need further investigation.

This study included genetic forms of dystonia, including *DYT-THAP1*, *DYT-TOR1A*, *DYT-SGCE*, *DYT-PRKRA*, PKAN, and Mohr–Tranebjaerg syndrome. *DYT-TOR1A*, *DYT-SGCE*, and *DYT-THAP1* are considered to be responsive to DBS, albeit to varying degrees [14,15]. Regarding *DYT-PRKRA* and Mohr–Tranebjaerg syndrome, there are few reports and case series showing a response to DBS [16,17]. However, more studies focusing on different genetic forms of dystonia are needed.

An interesting observation regarding the botulinum toxin dosage and DBS battery can be made in one of our cases. This particular patient experienced a marked increase in the BoNT dosage until battery replacement.

Our study has some limitations, such as its small sample size, non-blinded nature, patients with different types of dystonia and DBS targets, as well as the fact that the study is a retrospective analysis. In addition, examiner heterogeneity was mainly due to the different centers involved, which can lead to different protocols. Also, adjustments to DBS settings were made according to each patient’s symptoms, and not following a specific protocol, thus resulting in substantial variability between patients and centers. Furthermore, even though we used a literature-based dose conversion, different brands of toxins were used.

Another significant aspect to consider is if the BoNT dosage reduction impacted patients in a clinical manner. Patients usually have a motor improvement after DBS. This improvement varies and is influenced by a number of different factors regarding the type of dystonia, patient characteristics, lead location, and so forth. Thus, there are excellent, good, and poor responders to DBS. This motor improvement may thus lead to modified BoNT application (fewer muscles, less dosage, longer application intervals), which may reflect the motor improvement. This study aimed to evaluate if this is the case (with limitations). More studies, especially prospective studies, are needed to replicate our findings. Nevertheless, BoNT dose reduction implies symptom control improvements and lowers the costs for this specific treatment.

## 4. Conclusions

In conclusion, despite the limitations of this retrospective analysis, the study showed a statistically significant dose reduction in BoNT after DBS surgery in patients with generalized dystonia. This observation suggests that these treatments may have a combined effect. In addition, as shown in this study, it is important to recognize that certain body segments (axial muscles, for instance) may have a greater response after DBS, although this data needs further investigation.

Further prospective studies with specific treatment protocols, as well as long-term analyses, could improve our understanding of the role of BoNT combined with DBS in the management of generalized dystonia.

## 5. Materials and Methods

This was a retrospective study. The data was collected by reviewing the charts of dystonic patients with DBS at five different centers: Hospital das Clínicas da Universidade de São Paulo; Hospital Universitário Cajuru; University Health Network, Toronto Western Hospital; Instituto Neurológico de Curitiba; and Hospital Universitário Clementino Fraga Filho.

The study included twenty-three patients diagnosed with generalized dystonia who underwent DBS and received BoNT injections before surgery and at least 6 months after surgery. Patients with no available data on their charts or those who did not use BoNT prior to DBS were excluded. After data collection, an analysis of the 23 BoNT protocols before and after DBS was performed.

The injected muscles and BoNT doses were recorded for the last injection session prior to DBS and followed for at least 6 months from the date of surgery up to the last time point available. The primary outcome was to analyze the relationship between BoNT dosage and DBS surgery in each muscle. A secondary analysis of BoNT dosages through different muscle segments was performed (cervical, axial, orofacial, and appendicular).

Botulinum toxin dosage is expressed as median ± standard deviation. When onabotulinum toxin was used, a conversion rate of 1:3 (onabotulinum:abobotulinum) was used, as recommended in the literature, in order to perform a uniform analysis [18,19,20]. DBS settings were adjusted starting with lower electrical parameters on the best contacts predicted by monopolar review and image analysis. There was no standardized stimulation protocol among different centers and providers for subsequent adjustments. Thus, settings were adjusted based on individual clinical demands and were performed independent of BoNT injections or oral medication intake. Adjustments of DBS parameters, oral medications, and BoNT dosages were managed by the same center where surgery was performed.

Qualitative variables were described using absolute and relative frequencies. Quantitative variables are presented as median and interquartile ranges (IQR). Linear mixed models were used to compare toxin dosages at different time points. Using this model, contrasts between different moments of data collection were calculated. P-values were adjusted for multiple comparisons using the Bonferroni method. All analyses were performed using the R software 4.2.3, and the significance level was set at 0.05.

## Figures and Tables

**Table 1:** Baseline characteristics of the patients.

Patients	N = 23 ^1^
**Age**	35.00 (26.00–45.50)
**Etiology**	
DYT-*TOR1A*	2 (8.7%)
DYT-*THAP1*	2 (8.7%)
DYT-SGCE	1 (4.3%)
DYT-*PRKRA*	1 (4.3%)
Mohr–Tranebjaerg Syndrome	1 (4.3%)
Perinatal Hypoxia	1 (4.3%)
PKAN	1 (4.3%)
Tardive Dystonia	1 (4.3%)
**DBS Target**	
GPi	17 (74%)
STN	6 (26%)
**Surgery’s year**	
2008	1 (4.3%)
2011	1 (4.3%)
2012	1 (4.3%)
2016	1 (4.3%)
2017	6 (26%)
2018	5 (22%)
2019	2 (8.7%)
2020	2 (8.7%)
2022	4 (17%)

^1^ Median (AIQ); n (%).

**Table 2:** Total dose of botulinum toxin (U) per period.

	Dose pre, N = 23 ^1^	Dose 6 Months, N = 17 ^1^	Dose 12 Months, N = 21 ^1^	Current Dose, N = 20 ^1^
Values (UI)	800.0 (650.0–1294.0)	600.0 (300.0–804.0)	640.0 (375.0–900.0)	700.0 (300.0–1063.8)

^1^ Median (AIQ).

**Table 3:** Mean difference (dose in Units) per time.

Contrast	Mean Difference	CI 95%	*p* Value
Dose pre—6 months dose	293.4	114.3, 472.6	0.011
Dose pre—12 months dose	292.6	126.3, 458.9	0.006
Dose pre—Current dose	295.2	125.9, 464.5	0.006
6 months dose—12 months dose	−0.8	−185.3, 183.7	>0.999
6 months dose—Current dose	1.8	−184.0, 187.5	>0.999
12 months dose—Current dose	2.6	−171.8, 176.9	>0.999

**Table 4:** Mean difference (dose in Units) per period–splenius capitis muscle.

Contrast	Mean Difference	CI 95%	*p* Value
Dose pre—6 months dose	126.5	53.1; 199.8	<0.001
Dose pre—12 months dose	118.0	52.3; 183.7	<0.001
Dose pre—Current dose	119.1	54.4; 183.9	<0.001

**Table 5:** Mean difference (dose in Units) per period–paravertebral muscles.

Contrast	Mean Difference	CI 95%	*p* Value
Dose pre—6 months dose	106.6	−10.8; 224.0	0.087
Dose pre—12 months dose	124.0	25.3; 222.7	0.009
Dose pre—Current dose	135.8	39.3; 232.2	0.003

**Table 6:** Mean difference (dose in Units) per period–sternocleidomastoid muscle.

Contrast	Mean Difference	CI 95%	*p* Value
Dose pre—6 months dose	36.9	−32.4; 106.1	0.492
Dose pre—12 months dose	34.5	−26.3; 95.3	0.435
Dose pre—Current dose	38.8	−19.4; 96.9	0.295

**Table 7:** Mean difference (dose in Units) per period–semispinalis muscle.

Contrast	Mean Difference	CI 95%	*p* Value
Dose pre—6 months dose	4.7	−52.6; 62.0	0.996
Dose pre—12 months dose	−3.3	−58.1; 51.5	0.998
Dose pre—Current dose	−20.8	−71.4; 29.8	0.696

**Table 8:** Mean difference (dose in Units) per period–trapezius muscle.

Contrast	Mean Difference	CI 95%	*p* Value
Dose pre—6 months dose	17.4	−43.8; 78.5	0.872
Dose pre—12 months dose	14.8	−41.3; 70.9	0.895
Dose pre—Current dose	−14.6	−69.5; 40.4	0.894

## Data Availability

The raw data supporting the conclusions of this article will be made available by the authors on request.
